# Remarkable Case of Paraphymosis

**Published:** 1846-07

**Authors:** 


*Remarkable Case of Paraphymosis.*—Mr. Hancock was consulted on the 7th of January, 1846, by a gentleman aged sixty-five years, suffering from paraphymosis. The penis was much swollen, and the prepuce, especially at the under part, much thickened, attended with great pain. He stated, that for three months previously he had experienced sensations of tightness and heat round the cornea glandis, that the parts occasionally swelled, at times being better, at others worse. He described the sensation as that of a tightness, extending from the tip of the penis to the cornea glandis, underneath which it had increased rapidly for the last three weeks, so much so that he was induced to come to London from Suffolk, for advice. For the last two years he had ridden very spirited horses, and being a fat, heavy man, had frequently struck his penis against the saddle, but without at the time experiencing any inconvenience. Has suffered much pain latterly, as if a string were tied tightly round the cornea glandis; has had a double hernia, of large size, for several years, and has been in the habit of wearing trusses having strong heavy springs. His prepuce was not tight naturally.

Mr. Hancock endeavored to reduce the parts in the usual manner, and thought he had succeeded, but in two days the patient returned with the paraphymosis as bad as before, having suffered severely from the endeavors to remove it. Mr. Hancock having drawn back the skin, divided the thickened mucous membrane with a sharp-pointed bistoury—as was usually done in cases of paraphymosis—and desired him to go home, where he would visit him on the following day. On the 10th he again saw him, and found him as bad as before, not at all revived; the glans and the whole of the penis were swollen, the penis being twisted, inflamed, and exceedingly painful. He examined the parts very carefully; the skin was free, as was also the mucous membrane, which had been completely divided by the cut made yesterday, but he found a band of condensed cellular tissue, about a quarter of an inch wide, encircling the cornea glandis, and which he concluded was the cause of all the mischief; he consequently cut down through this to the fibrous covering of the corpus cavernosum, with immediate and decided relief to the patient, who is now nearly well, although thickening of the prepuce still exists.

Mr. Hancock related this case to the Westminster Medical Society as presenting some points of interest; the paraphymosis was not the result of any gonorrhœal discharge, or sores on the glans, its production was neither preceded nor attended, until quite latterly, by any active inflammation, but the mischief apparently was produced by gradual thickening and induration of the cellular tissue surrounding the cornea glandis, probably aggravated by the constant dragging exercised on the parts by the strong truss he was in the habit of wearing. Another point worthy of notice was, that in the common forms of paraphymosis the result of acute inflammation of the prepuce, originally too contracted, the stricture was situated in the mucous membrane lining the prepuce, and the mischief was at once removed by dividing it with a bistoury from within outwards, without cutting through the integuments, but in the case here related, the mere division of the mucous membrane, as commonly performed, was attended by no benefit whatever, as it was not until the subsequent day, when he cut down on the cornea glandis to the fibrous covering of the penis, that his patient was at all revived.

Within the last few months, he had been consulted by two other gentlemen for spontaneous thickening and induration of the prepuce, for which they could assign no cause. In one it induced phymosis, in the other paraphymosis. In the latter he cut the mucous membrane, but as he never saw the patient again, he had no means of knowing the result. However, the case here related showed that in spontaneous paraphymosis the stricture may depend upon thickening cellular tissue, and not upon any change in the mucous membrane.—*Ibid.*

